# Hyperthyroidism in pregnancy: design and methodology of a Danish multicenter study

**DOI:** 10.1186/s13044-023-00154-8

**Published:** 2023-04-14

**Authors:** Nanna Maria Uldall Torp, Inge Bülow Pedersen, Allan Carlé, Jesper Scott Karmisholt, Eva Ebbehøj, Diana Grove-Laugesen, Thomas Heiberg Brix, Steen Joop Bonnema, Bieke F. Schrijvers, Birte Nygaard, Lena Bjergved Sigurd, Ulla Feldt-Rasmussen, Marianne Klose, Åse Krogh Rasmussen, Stig Andersen, Stine Linding Andersen

**Affiliations:** 1 Department of Clinical Biochemistry, Aalborg University Hospital, Hobrovej 18-22, Aalborg, 9000 Denmark; 2 Department of Clinical Medicine, Aalborg University, Aalborg, Denmark; 3 Department of Endocrinology, Aalborg University Hospital, Aalborg, Denmark; 4 Department of Endocrinology and Internal Medicine, Aarhus University Hospital, Aarhus, Denmark; 5 Department of Endocrinology, Odense University Hospital, Odense, Denmark; 6 Institute of Clinical Research, University of Southern Denmark, Odense, Denmark; 7 Department of Endocrinology, Zealand University Hospital Køge, Køge, Denmark; 8 Department of Endocrinology, Copenhagen University Hospital Herlev-Gentofte, Herlev, Denmark; 9 Department of Clinical Medicine, Copenhagen University, Copenhagen, Denmark; 10 Department of Endocrinology and Metabolism, Copenhagen University Hospital Rigshospitalet, Copenhagen, Denmark; 11 Deparment of Geriatrics, Aalborg University Hospital, Aalborg, Denmark

**Keywords:** Gestation, Antithyroid, Methimazole, Propylthiouracil, Levothyroxine, Graves’ disease

## Abstract

**Background:**

Graves’ disease (GD) is the main cause of hyperthyroidism in women of the fertile age. In pregnant women, the disease should be carefully managed and controlled to prevent maternal and fetal complications. Observational studies provide evidence of the adverse effects of untreated hyperthyroidism in pregnancy and have in more recent years substantiated a risk of teratogenic side effects with the use of antithyroid drugs (ATDs). These findings have challenged the clinical recommendations regarding the choice of treatment when patients become pregnant. To extend observational findings and support future clinical practice, a systematic collection of detailed clinical data in and around pregnancy is needed.

**Methods:**

With the aim of collecting clinical and biochemical data, a Danish multicenter study entitled ‘Pregnancy Investigations on Thyroid Disease’ (PRETHYR) was initiated in 2021. We here describe the design and methodology of the first study part of PRETHYR. This part focuses on maternal hyperthyroidism and recruits female patients in Denmark with a past or present diagnosis of GD, who become pregnant, as well as women who are treated with ATDs in the pregnancy, irrespective of the underlying etiology. The women are included during clinical management from endocrine hospital departments in Denmark, and study participation includes patient questionnaires in pregnancy and postpartum as well as review of medical records from the mother and the child.

**Results:**

Data collection was initiated on November 1, 2021 and covered all five Danish Regions from March 1, 2022. Consecutive study inclusion will continue, and we here report the first status of inclusion. As of November 1, 2022, a total of 62 women have been included in median pregnancy week 19 (interquartile range (IQR): 10–27) with a median maternal age of 31.4 years (IQR: 28.5–35.1). At inclusion, 26 women (41.9%) reported current use of thyroid medication; ATDs (n = 14), Levothyroxine (n = 12).

**Conclusion:**

This report describes a newly established systematic and nationwide collection of detailed clinical data on pregnant women with hyperthyroidism and their offspring. Considering the course and relatively low prevalence of GD in pregnant women, such nationwide design is essential to establish a sufficiently large cohort.

## Introduction

The most common cause of hyperthyroidism in women of the fertile age is Graves’ disease (GD) [1], and the management of the disease in this patient group includes the possibility of a future pregnancy. It is well-established that GD in pregnant women should be carefully managed and controlled to prevent maternal and fetal complications, and preconception counseling is recommended [2, 3]. This counseling prior to pregnancy should consider disease activity as well as treatment options before and during a pregnancy [2, 3]. The management of hyperthyroidism in women with GD is challenged in pregnancy because of concerns about thyroid function of the pregnant woman and the fetus [4, 5]. This is reflected by recommendations on tight biochemical monitoring, and avoidance of treatment strategies that may harm the fetus [2, 3]. However, uncertainties regarding the management of GD in pregnant women exist [2, 3].

The treatment of choice for the hyperthyroidism of GD in pregnancy is antithyroid drugs (ATDs) [6], and the available ATDs include Methimazole (MMI), its prodrug Carbimazole (CMZ), as well as Propylthiouracil (PTU) [7]. The use of ATDs is not without concern, as they may be associated with side effects [7–9]. A pregnancy-specific concern is the risk of teratogenic side effects, especially in early pregnancy when the fetal organs develop [10]. A risk of birth defects associated with MMI/CMZ has long been considered and substantiated in observational studies; however, a teratogenic effect of PTU has also been brought forward [10]. This potential teratogenic side effect of all available ATDs, the lack of alternative treatments, and the known adverse effects of uncontrolled maternal hyperthyroidism challenge the treatment recommendations in and around early pregnancy [2, 3]. Similarly, more evidence is needed to support future clinical practice regarding the recommendations for the choice of treatment in later pregnancy and the biochemical monitoring of maternal and fetal thyroid function, including the concentration of thyroid-stimulating hormone (TSH)-receptor antibodies (TRAb) [2, 3].

Large, observational studies provide unique opportunities to study relatively rare disorders and outcomes. However, such studies are often limited by being register-based with an indirect assessment of exposure from diagnoses and redeemed prescriptions of drugs [11]. To extend the register-based findings and support future clinical practice on the management of GD in pregnant women, detailed clinical data on treatment and biochemical monitoring are needed. We here present the design and methodology of a newly established Danish nationwide multicenter study on pregnant women with GD and women treated with ATDs in the pregnancy irrespective of the underlying etiology, in which detailed clinical and biochemical data are collected from mother and child during pregnancy and follow-up.

## Materials and methods

**Fig. 1:**
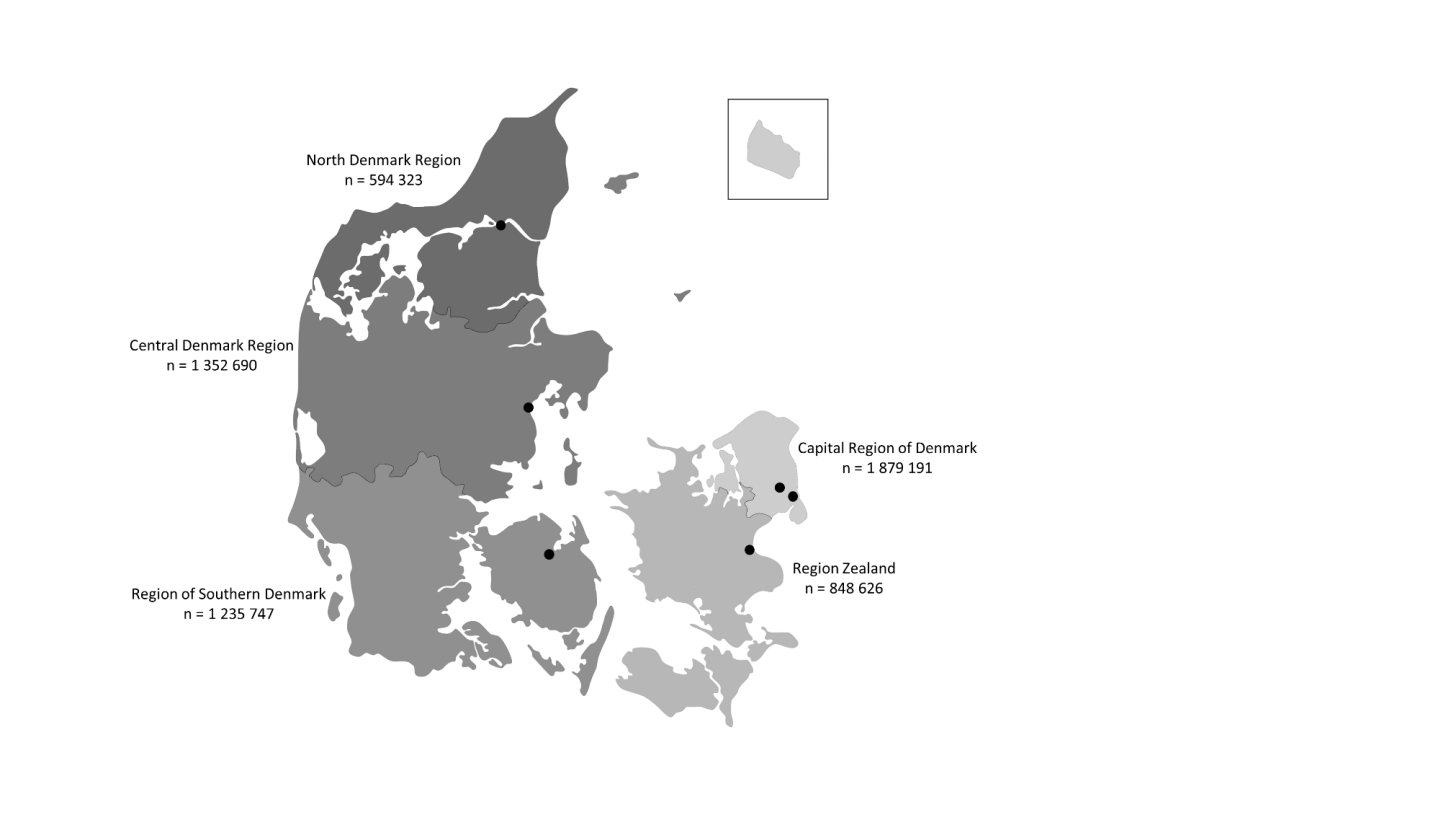
Overview of the Danish regions and participating hospitals.

An observational nationwide, multicenter study entitled ‘Pregnancy Investigations on Thyroid Disease’ (PRETHYR) was established in Denmark in 2021. The first part of PRETHYR focuses on maternal hyperthyroidism in pregnancy and aims to collect systematic, detailed clinical and biochemical data on women in Denmark with GD who become pregnant as well as women with hyperthyroidism of other etiologies treated with ATDs in pregnancy. In Denmark, the management of pregnant women with thyroid disease is a specialist function and it is a strong recommendation that these patients are referred from general practice to hospital management when pregnancy is planned or as soon as it is detected. The management of pregnant women with GD is carried out in the endocrine departments across the five Danish regions (Fig. 1). The PRETHYR study is designed as a multicenter study with participating centers at the Department of Endocrinology, Aalborg University Hospital (the North Denmark Region), Department of Endocrinology, Aarhus University Hospital (Central Denmark Region), Department of Endocrinology, Odense University Hospital (Region of Southern Denmark), Department of Endocrinology, Zealand University Hospital Køge (Region Zealand), and Departments of Endocrinology at Copenhagen University Hospital Herlev-Gentofte and at Copenhagen University Hospital Rigshospitalet (Capital Region of Denmark) (Fig. 1).

The study is anchored at Aalborg University Hospital, where a research group coordinates ethical permissions, study materials, and data collection. The overall aim is to recruit all pregnant women with GD and all women treated with ATDs in pregnancy from the participating endocrine departments. Specifically, the study recruits all pregnant women above the age of 18, who have a prior, current, or newly defined diagnosis of GD, irrespective of the current disease activity and treatment. Newly diagnosed GD in the pregnancy is defined as suppressed TSH in combination with TRAb above the method-specific cut-off used in the clinical laboratory [2, 3]. Furthermore, pregnant women with hyperthyroidism of other causes than GD are included if the woman is treated with ATDs in the current pregnancy. It was an *a priori* decision to not include women with gestational hyperthyroidism as treatment with ATDs is not recommended for the management of this disorder [3]. The women are enrolled during the first visit to the endocrine department after a pregnancy is detected. Thus, the project-responsible doctors in the participating endocrine departments identify the women eligible for enrollment and obtain the women’s consent to be contacted for further information about the study. If consent to contact is obtained, the woman is contacted by the study coordinator at Aalborg University Hospital, who informs about the study and asks for informed consent.

**Fig. 2:**
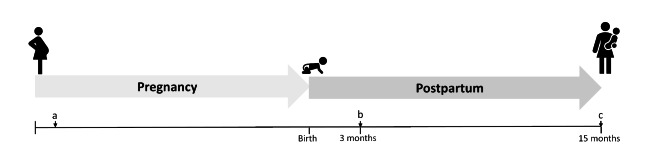
Overview of the study timeline: a) inclusion and index questionnaire, b) first review of medical records, postpartum inclusion, and postpartum questionnaire, and c) second review of medical records.

Upon informed consent to participate, the study is carried out in two parts for each participant (Fig. 2); the pregnancy part and the postpartum part, and the primary data sources are patient questionnaires and medical records. At study inclusion in the pregnancy, the woman is asked to fill out an electronic questionnaire (Fig. 2, time point a). Furthermore, the father of the child is invited to fill out a questionnaire. Three months after the completion of the pregnancy, the woman is asked for informed consent to participate in the postpartum part of the study. Upon informed consent, the woman is again asked to fill out an electronic questionnaire (Fig. 2, time point b). All questionnaires are followed by a telephone interview to verify the answers and clarify any uncertainties. In parallel with the patient questionnaires, medical records of the woman and her child are collected three months and 15 months postpartum (Fig. 2, time points b and c). All questionnaires are written in Danish, however, for non-Danish-speaking participants the questionnaire is carried out by a telephone interview in English.

Detailed information on the mother, father, and child is collected from the various data sources (Table 1). Thus, information not necessarily reported as standard in the medical record can be obtained from the questionnaire and vice versa. Furthermore, this collection of data from different sources allows for comparison of the information gathered to increase data validity. Specifically, detailed data are collected from both data sources on maternal use of ATDs in pregnancy including any timing of a shift in therapy. The questionnaire holds detailed questions on maternal lifestyle factors and current use of dietary supplements including iodine-containing supplements. To cover maternal thyroid symptoms at the time of answering the questionnaires, specific questions concerning hyperthyroid symptoms were excerpted from the ThyPRO questionnaire [12]. An important focus in the postpartum data collection is any adverse effects of maternal hyperthyroidism or the treatment concerning mother and child. Specifically, medical records are reviewed for any maternal complications at birth of the child, and child records are reviewed for complications and any diagnosis of birth defects. Since less severe birth defects are not necessarily detected at birth and diagnosed in a hospital, the questionnaire to the mother postpartum includes questions on child health including any disease or birth defect in the child (Table 1).

**Table 1:** Overview of data collected in the study. The maternal medical record was reviewed three months postparum (medical record 1) and fifteen months postpartum (medical record 2).

	Mother				Child	Father
	Index questionnaire	Medical record 1	Postpartum questionnaire	Medical record 2	Medical record	Questionnaire
**Main characteristics**						
Sociodemographics	X	X	X	X		X
Lifestyle factors	X	X	X	X		X
Health and comorbidity	X	X	X	X	X	X
Medication use	X	X	X	X	X	X
Dietary supplement use	X		X			X
Reproductive history	X	X	X	X		
Fetal health		X				
Child health			X		X	
**Thyroid characteristics**						
Family history	X					X
Disease history	X	X			X	X
Symptoms	X	X	X	X	X	X
Biochemical assessments		X		X	X	
Other examinations (ultrasound, scintigraphy)		X		X	X	

Study inclusion was initiated on November 1, 2021, with participation from all five Danish regions as of March 1, 2022. Data inclusion will proceed continuously for an initial planned period of two years. Information from the questionnaires and medical records are collected and managed using Research Electronic Data Capture (REDCap) [13, 14] and hosted at Aalborg University Hospital. The Committee on Health Research Ethics deemed the study exempt from review. The study is registered according to the General Data Protection Regulation in the North Denmark Region (2021 − 166), and collaboration agreements for data transfer across the regions have been obtained. All data are analyzed in encrypted form so that no individuals can be identified by the researchers, and participation in the study has no impact on the management of maternal or child disease in the current pregnancy.

Statistical analyses were performed using STATA 17 (StataCorp, Texas).

## Results

As of November 1, 2022, 74 pregnant women were recruited from the endocrine departments in Denmark, of whom a total of 62 women (83.8%) agreed to participate in the study. The women who were recruited, but not included, either never answered the phone or decided not to participate in the study after further information. At the time of answering the index questionnaire in pregnancy (Fig. 1, time point a), the women included were median in the 19th week of pregnancy (18 + 0 to 18 + 6 full weeks + days) with interquartile range (IQR) from the 10th to the 27th week of pregnancy. The pregnant women had a median age of 31.4 years (IQR: 28.5–35.1) at inclusion, and six women (9.7%) described themselves as being of non-Danish origin. The median pre-pregnancy body mass index (BMI) of the women was 23.8 kg/m^2^ (IQR: 21.3–27.5 kg/m^2^), and 18 women (29.0%) were current smokers or had a history of smoking. Regarding reproductive history, 32 women (51.6%) were nulliparous, and 13 (21.0%) reported having received fertility treatment as part of the pregnancy under study.

The women were median 28.0 years (IQR: 25.0–32.0 years) at the time when hyperthyroidism was first diagnosed. Most of the women (88.7%) had preexisting hyperthyroidism at the time of study inclusion which was diagnosed median 2.9 years (IQR: 1.1–5.1 years) before the current pregnancy. Of these women with a prior thyroid diagnosis, 38 (69.1%) were referred to and managed by a specialist in the endocrine department at the time of becoming pregnant. A total of seven women (11.3%) were first diagnosed with hyperthyroidism during the current pregnancy. Regarding medical treatment of the hyperthyroidism in the pregnancy, a total of 14 women (22.6%) reported the use of ATDs at inclusion, 12 women (19.4%) were treated with Levothyroxine, and none of the women received combination therapy with ATDs and Levothyroxine. Among these 12 women treated with Levothyroxine, a total of 3 women (25%) had received prior definitive treatment. Among the women treated with ATDs, five women (35.7%) reported a use of MMI, and 9 women (64.3%) reported a use of PTU.

As of November 1, 2022, sixteen of the 62 women included have completed their pregnancy and have been invited to participate in the postpartum follow-up three months after the birth of the child. Among these women invited postpartum, 14 (87.5%) have agreed to participate.

## Discussion

This is the first report on a newly established Danish nationwide collection of clinical data on pregnant women with a previous history of or current GD and hyperthyroid women treated with ATDs in pregnancy, irrespective of the underlying etiology. We describe the study design, the method of data collection, and the initial report on the number of women included. The study aims to collect systematic and detailed clinical data regarding diagnosing, monitoring, and treating hyperthyroidism in pregnancy, as well as the outcome of mother and child within this patient group to extend the previous observational findings and support future clinical practice.

Hyperthyroidism in pregnant women has long been a matter of concern [15]. Evidence to support the adverse effects of uncontrolled hyperthyroidism in pregnant women with GD dates nearly a century back to reports in the 1920s to 1940s, which was before the development of ATDs, illustrating the severe consequences of the disease for mother and child when left untreated during pregnancy [16]. Since then, ATDs have been developed [17] and used as the recommended treatment of maternal hyperthyroidism in pregnancy and it is unanimously stated in clinical guidelines that GD in women who become pregnant should be carefully monitored and controlled to prevent complications [2, 3]. Even though the indication for treatment and the available treatments have been part of clinical practice for decades, uncertainties regarding clinical practice prevail as reflected by weak evidence to support some recommendations [2, 3]. Some of the knowledge gaps identified and discussed in current clinical guidelines include aspects of treatment with ATDs, especially in and around the early pregnancy as well as monitoring of maternal hyperthyroidism and fetal thyroid status in the later pregnancy [2, 3]. Since the earliest reports illustrating the adverse effects of severe and untreated maternal hyperthyroidism in pregnancy, most studies have been observational register-based studies [15]. The advantage of the register-based studies is that they are large and nationwide, which is important when studying a relatively rare exposure and likewise rare outcomes. The expected prevalence of hyperthyroidism caused by GD in pregnant women is 0.2% [2, 3], implying that, a substantial number of pregnant women need to be observed to obtain robust findings. Furthermore, the nationwide design and the use of retrospective register-data reduce the risk of selection bias. Adding to this, the large observational studies published within the last decades have substantiated the risk associated with maternal hyperthyroidism and the treatment by the study inclusion of non-exposed control groups [10], and extended the initial case series on the adverse effects of maternal hyperthyroidism and the risk of teratogenic side effects associated with the treatment [18].

Overall, the large observational studies conducted in different populations within the last decade have corroborated a risk of birth defects with the use of MMI/CMZ showing that these side effects are rare, but often severe [10]. This finding has led to the recommendation of using PTU in early pregnancy and the suggestion that women who receive MMI/CMZ could be shifted to PTU when a pregnancy is planned or as soon as it is detected [2, 3]. However, an increased risk of birth defects after exposure to PTU has also been brought forward, and even if these malformations appear less severe, an association has been observed across different study populations [19, 20]. This finding has challenged the clinical guidance as to which drug to use and proposals have been made regarding alternative treatments or withdrawal of treatment during the critical weeks of early pregnancy in appropriately selected patients [18]. To extend knowledge and support future clinical practice, a focus on the disease characteristics and outcome of women who shift or withdraw therapy in and around early pregnancy is warranted. Again, a large study cohort is preferred, however, the limitation of observational register-based studies is that the exposure is indirectly defined meaning that diagnoses of disease, registrations of hospital contacts, and redeemed prescriptions of drugs are used to define maternal hyperthyroidism and the treatment in the pregnancy. Even if the register-data are validated for research purposes, it is difficult to obtain detailed information on the exact timing of a shift in therapy for example [21]. Another determinant is the role of maternal thyroid function and thyroid autoimmunity *per se* in the associations observed [10]. Regarding maternal hyperthyroidism in early pregnancy, it is crucial to differentiate between GD and gestational hyperthyroidism caused by the effect of human chorionic gonadotropin [22]. Even if pregnancy-specific reference ranges for maternal TSH are applied, most women identified with low TSH in early pregnancy are likely to suffer from gestational hyperthyroidism [23]. Accordingly, detailed clinical information including results of the measurement of TRAb, are warranted to identify pregnant women with hyperthyroidism caused by GD in observational outcome studies.

To extend the observational register-based findings on maternal hyperthyroidism in pregnancy, we designed and initiated the nationwide, multicenter study ‘PRETHYR’ presented in this report. Our study focus is on hyperthyroidism in pregnant women and outcomes of treatment with ATDs, and not on gestational hyperthyroidism. Thus, it was an *a priori* decision to focus study inclusion on pregnant women with GD, which is the predominant cause of hyperthyroidism among fertile women in Denmark [1]. In addition, we include pregnant women treated with ATDs for hyperthyroidism of other etiologies to study all pregnancies exposed to ATDs. The combined data collection via patient questionnaires and review of medical records of mother and child allows for detailed assessment of biochemical analyses of thyroid function and thyroid auto-antibodies as well as information on treatment in and after the pregnancy. Furthermore, detailed information on any adverse outcomes of pregnancy or fetal development will be registered with information on birth defects from the medical records as well as from maternal self-report. Data sources and timing of assessment for birth defects are some proposed explanations for the divergent findings across observational studies regarding the association between ATDs and birth defects [10, 11]. Study inclusion in ‘PRETHYR’ has now been ongoing for about a year. One of the challenges with the establishment of a nationwide, multicenter data collection was data rules and regulations across the different regions of Denmark and the process and time frame of obtaining collaboration agreements. This issue explains the stepwise study initiation in the participating hospitals. With approximately 60,000 births in Denmark per year [24], we *a priori* expect to include about 120 (0.2%) pregnant women with GD per year. This relatively low figure highlights the importance of nationwide data collection to obtain enough exposed cases, however, even though Denmark is a rather small country of 5,910,577 inhabitants [25], each geographical region has its own healthcare administration and computerized health record system which may change over time. Thus, a systematic data collection must ensure that data from the different electronic health record systems can be collected in REDCap and that historical data can be obtained when a change in the electronic system is implemented. Regarding the biochemical results collected in the study database, evaluation of results will be performed according to the local assay used. Thus, information will be gathered on assay-specific reference ranges and assay specific performance. The present study is anchored at the large university hospitals in each of the five Danish regions which hold specialized units for the management of thyroid disease in pregnant women. The study design ensures a nationwide inclusion and a high rate of participation among invited women; however, we cannot exclude that some women eligible for inclusion are managed during pregnancy at smaller regional hospitals without referral to specialized units. The study is observational, and data are collected according to current clinical care and not from a protocolled design. However, nationwide recommendations for the clinical management of thyroid disease in pregnant women are implemented in Denmark which supports equal patient management across regions. Finally, the inclusion of information from the fathers is a challenge because data regulations define that the fathers can only be invited for participation via the participating pregnant woman.

## Conclusions and perspectives

This report presents the aim, design, and establishment of a Danish nationwide multicenter collection of detailed clinical data on pregnant women with GD as well as pregnant women with hyperthyroidism of other etiology who are treated with ATDs. Study inclusion has been ongoing for about a year and will proceed continually. We describe a setup ensuring nationwide data collection that works across regions and hospitals which is deemed necessary to obtain adequate numbers of exposed cases. The study design and methodology may be applicable to other countries to increase cohort size and generalizability of the results.

## Data Availability

The data generated and analyzed during the current study are not publicly available to ensure individual confidentiality but can be made available from the corresponding author upon reasonable request.
